# Personalized cognitive-behavioural therapy for obesity (CBT-OB): theory, strategies and procedures

**DOI:** 10.1186/s13030-020-00177-9

**Published:** 2020-03-09

**Authors:** Riccardo Dalle Grave, Massimiliano Sartirana, Simona Calugi

**Affiliations:** 1 Department of Eating and Weight Disorders, Villa Garda Hospital, Via Monte Baldo 89 37016 Garda (VR), Verona, Italy; 2 Associazione Disturbi Alimentari, Via Sansovino 16, 37016 Verona, Italy

**Keywords:** Obesity, Treatment, Cognitive-behavioural therapy, Outpatient, Day-hospital, Residential treatment

## Abstract

Personalized cognitive-behavioural therapy for obesity (CBT-OB) is a new treatment that combines the traditional procedures of standard behavioural therapy for obesity (i.e., self-monitoring, goal setting, stimulus control, contingency management, behavioural substitution, skills for increasing social support, problem solving and relapse prevention) with a battery of specific cognitive strategies and procedures. These enable the treatment to be individualized, and to help patients to address the cognitive processes that previous research has found to be associated with treatment discontinuation, the amount of weight lost and long-term weight-loss maintenance. The treatment programme can be delivered at three levels of care, outpatient, day hospital and residential, and includes six modules, which are introduced according to the individual patient’s needs as part of a flexible, personalized approach. The primary goals of CBT-OB are to help patients to (i) achieve, accept and maintain healthy weight loss; (ii) adopt a lifestyle conducive to weight control; and (iii) develop a stable “weight-control mindset”. A randomized controlled trial has found that 88 patients suffering from morbid obesity treated with CBT-OB followed a period of residential treatment achieved a mean weight loss of 15% after 12 months, with no tendency to regain weight between 6 and 12 months. The treatment efficacy is also supported by data from a study assessing the effects of group CBT-OB delivered in a real-world clinical setting. In that study, 77 patients with morbid obesity who completed the treatment achieved 9.9% weight loss after 18 months. These promising results, if confirmed by future clinical studies, suggest that CBT-OB has the potential to be more effective than traditional weight-loss lifestyle-modification programmes.

## Background

The key strategy of obesity management is to create a negative energy balance that allows patients to attain a healthy weight. This is usually achieved by combining specific recommendations about diet and exercise—a lifestyle modification strategy that is successful in producing some degree of weight loss in many patients. However, in order to avoid weight regain, patients need to maintain a persistent neutral energy balance, and many treatments for obesity fail in this regard, making them ineffective in the long term.

The failure of weight-loss treatments based on lifestyle modification is generally ascribed to powerful biological pressures causing patients with obesity to overeat; it is difficult for such patients to resist the pressure to regain the weight they have lost, especially if they are surrounded by tasty, calorie-rich foods and rely on labour-saving devices on a daily basis [1]. This, however, does not explain why some individuals are able to stay ‘on the wagon’, persevering with the weight-control techniques they have learned and thereby preventing weight regain in the long term [2, 3]. It has been postulated that there are specific cognitive processes acting in these individuals, helping them maintain long-term adherence to lifestyle modification [4].

However, the treatments traditionally offered to patients with obesity mainly aim to counter the biological and behavioural factors hindering weight loss and maintenance, with very little regard to the cognitive processes that may be at play. Failure to address a patients’ ability to adhere to lifestyle modification over time may therefore be one of the reasons why biological and behavioural treatments have limited effectiveness in promoting long-term weight loss [5]. This theory has been lent weight by recent indications that there are several cognitive factors associated with treatment attrition, weight loss and weight maintenance, respectively [4], suggesting that success rates may be improved by developing new treatments to address these cognitive processes; even existing treatments for obesity could be enhanced by integrating specific cognitive strategies and procedures.

With this in mind, we describe here the theory and main strategies and procedures of a new cognitive behavioural therapy for obesity (CBT-OB), a treatment designed to help patients to achieve and maintain a healthy weight loss through personalized combinations of strategies and procedures from traditional behavioural therapy for obesity (BT-OB) with others addressing some specific cognitive processes that the evidence suggests can influence attrition, weight loss and weight-loss maintenance.

### Cognitive processes associated with attrition, weight loss and weight maintenance

Basic research clearly indicates that cognitive processes play an important role in maintaining excessive and dysregulated eating habits, making healthy eating difficult [6]. Moreover, several clinical studies in real-world settings have found significant associations between specific cognitive factors and treatment attrition, as well as the amount of weight patients are able to lose and maintain (Table 1) [14]. These findings have been the basis upon which CBT-OB has been designed, with a view to overcoming some of the shortcomings of traditional behavioural therapy for obesity (BT-OB) [15].

**Table 1:** Specific cognitive factors associated with treatment discontinuation, amount of weight lost and weight-loss maintenance

*Cognitive factors associated with treatment discontinuation*:	
• Higher expected 1-year BMI loss at baseline [7, 8]	
• Primary goal for weight loss based on appearance at baseline [8]	
• Acceptable or disappointing weight with respect to personal expectations [9]	
• Dissatisfaction with weight loss obtained through treatment [10]	
*Cognitive factors associated with amount of weight lost*:	
• Increase in dietary restraint and reduction in disinhibition [11]	
• Higher expected weight loss at baseline [12]	
*Cognitive factors associated with weight-loss maintenance*:	
• Satisfaction with the results achieved [7]	
• Weight-loss satisfaction [12]	
• Confidence in the ability to lose additional weight without professional help [7]	
• Greater weight-loss satisfaction from week 15 or 19 of the weight-loss phase (a decline is associated with weight regain) [13]	

From Dalle Grave et al. [15] p. 9. Reprinted with the permission of Springer Nature

### From BT-OB to CBT-OB

BT-OB was originally based on learning theory (i.e., behaviourism), and the idea that education, and the recognition and modification of environmental stimuli (antecedents) and consequences of food intake (reinforcements), can prompt patients to change their dietary and physical activity habits with a view to reaching and maintaining a healthy weight [7, 14] . The treatment was subsequently integrated with social cognitive theory (e.g., goal setting, modelling and self-efficacy) [8] and basic cognitive strategies (e.g., problem solving and cognitive restructuring) [9], as well as specific recommendations on diet and exercise [10]. BT-OB is an effective means of helping some patients achieve weight loss in the short term; after one year, about 25 and 30% manage to lose and maintain 5–9.9% and ≥ 10% of their body weight, respectively [11] with a mean drop-out rate of about 20% [12]. This rate of weight loss is associated with a significant reduction in the incidence of type 2 diabetes, not to mention improvements in other weight-related medical comorbidities, psychosocial problems and quality of life [13].

However, patients tend to achieve peak weight loss at around six months, and at five-year follow-up roughly 50% of BT-OB patients have returned to their original weight [16]. Indeed, even in its latest iteration, BT-OB is poorly individualized; it is generally administered to groups, and there is a prescribed order of sessions to follow, irrespective of each patient’s actual progress [5]. This may be due to the main goal of the treatment, helping patients achieve behavioural change (i.e., of eating healthily and exercising), falling short. With this in mind, we have developed a new form of treatment, integrating strategies and procedures that can help to bring about cognitive change—the aim of standard CBT [5]—and thereby improve long-term outcomes.

**Fig. 1:**
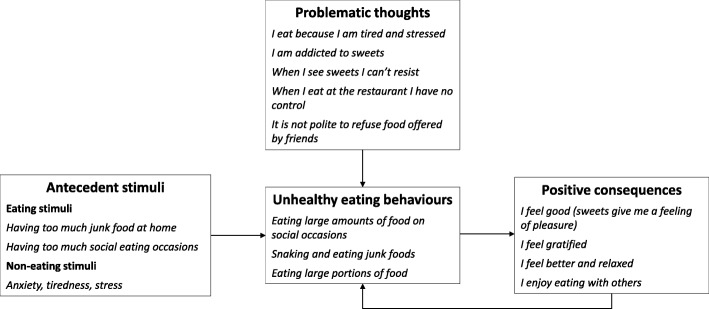
An example personal formulation featuring a patient’s main obstacles to weight loss

To this end, CBT-OB involves not only the four main core strategies of BT-OB [15], namely (i) using a specific model of the disorder maintenance; (ii) actively involving patients with a collaborative therapeutic style; (iii) using a problem-solving approach focused on the present; and (iv) assessing treatment efficacy and updating the strategies and procedures involved in response to clinical and research findings, but also integrates CBT-based strategies and procedures. In short, CBT-OB differs from BT-OB in the following key aspects [15]: (i) rather than solely pursuing behavioural change, it aims to produce cognitive change to influence the long-term maintenance of lifestyle modification; (ii) it is based on a personalized “cognitive conceptualisation”, also called a “personal formulation” (see Fig. 1), of the main mechanisms known to negatively influence weight loss and maintenance, tackling them by means of specific cognitive-behavioural strategies and procedures introduced according to the needs of the individual patient; (iii) it can be used to treat even patients with severe obesity and disability (usually not treated with standard BT-OB) via the adoption of intensive forms of the treatment (e.g., residential programmes).

### Goals, general strategies and procedures of CBT-OB

The main goals of CBT-OB are to help patients to (i) reach, accept and maintain a healthy amount of weight loss (i.e., 5–10% of their starting body weight) [13]; (ii) adopt and maintain a lifestyle conducive to weight control; and (iii) develop a stable “weight-control mind-set”. CBT-OB therapists adopt a therapeutic style designed to develop and nurture a collaborative working relationship (the therapist and patient(s) work together as a team). The treatment combines specific recommendations for a patient’s diet and exercise habits with procedures from both behavioural and cognitive forms of therapy. In addition to some of the procedures adopted by BT-OB (i.e., self-monitoring, goal setting, stimulus control, contingency management, behavioural substitution, skills for increasing social support, problem solving and relapse prevention) [17], the treatment includes specific cognitive strategies and procedures, some of which have been adapted from ‘enhanced’ CBT (CBT-E) for eating disorders [18] (i.e., engaging patients to make the treatment a priority and to play an active role in changing their own habits; organizing the agenda of the sessions; self-monitoring in real time; doing the strategically planned “homework; establishing a pattern of regular eating; identifying setbacks and respond promptly to them), and Cooper et al. CBT [5] (i.e., drawing distinction between weight loss and weight maintenance; addressing during the weight loss phase potential obstacles to the acceptance of weight maintenance such as unrealistic weight goals, primary goals, and body image concerns), and some of which have been developed by our team. However, CBT-OB differs from Copper et al. CBT [5] in the following main aspects: (i) it actively involves, with the patients’ agreement, significant other(s) to creating an environment that promotes positive changes in eating and physical activity habits; (ii) it provides patient a structured meal plan based on the food exchange lists; (iii) it trains patients to assess their energy expenditure and to develop not only an active lifestyle, but also an improvement of their physical fitness; (iv) it develops collaboratively with the patients their personal formulation of the processes that are hindering weight loss; (v) it encourage patients to fill in weekly the Weight-Loss Obstacles Questionnaire to identify not only the behaviours but also the cognitive processes that might hinder weight loss; (vi) it has a longer maintenance phase (48 weeks vs. 24 weeks); and (vii) it includes two intensive steps of care (i.e., day-hospital and residential CBT-OB) for patients with severe and disabling obesity. These integrations enable the treatment to be personalized, and help patients address with specific strategies and procedures the processes that our previous research has found to be respectively associated with drop-out, the amount of weight lost, and maintaining a lower weight in the long term (see Table 2).

**Table 2:** CBT-OB strategies and procedures for minimising attrition, enhancing weight loss and improving weight-loss maintenance

*Strategies and procedures for minimising attrition:*	
• Addressing patient’s difficulties attending the sessions	
- Scheduling the sessions at times compatible with a patient’s work commitments	
- Routinely asking the patients whether they are experiencing any difficulties as regards attending the sessions, and devoting time to understanding and/or overcoming them.	
• Showing interest in each patient as a person, irrespective of their weight and/or other issues	
- Adopting a “people first” policy—putting individuals before the disability or disease when describing persons affected by obesity (e.g., “person with obesity” instead of “obese person”	
- Avoiding any use of potentially pejorative adjectives or adverbs, or any language that implies moral judgements or highlights patients’ “character flaws” regarding their weight	
• Addressing unrealistic weight loss expectations	
- Encouraging patients to pursue and be satisfied with achievable short-term weight-loss goals (i.e., a weight loss of between 0.5 kg and 1.0 kg/week) and not disputing unrealistic goals at the beginning of treatment	
- Addressing unrealistic goals only when patients have achieved some success in reaching a healthy weight, but manifest dissatisfaction with the weight loss achieved	
• Maintaining therapeutic momentum	
- Identifying with the patients the best time to start the treatment	
- Stressing the importance of avoiding any interruptions in treatment, especially during the first 8 weeks	
- Explaining to the patients in advance that another therapist will take the place of the primary therapist in the event of their absence	
• Developing a protocol for dealing with late attendance or non-attendance	
- Encouraging patients to arrive a little early for session (e.g., 10–15 min) in order to relax and mentally prepare themselves	
- If patients are running late for an appointment, calling them after 15 min to express concern about their absence, and to try to reschedule the appointment as soon as possible	
*Strategies and procedures for enhancing weight loss*	
• Increasing dietary restraint and decreasing dietary disinhibition	
- Eating regularly (i.e., three planned meals and two snacks, and refraining from eating in the intervals between)	
- Planning meals in advance (when, what and where to eat) on a specific monitoring record, making reference to a structured meal plan	
- Supplying patients with grocery lists, menus and recipes	
- Monitoring food intake in real time	
- Training patients to eat consciously (i.e., “think while you are eating”)	
- Training patients to “ride out” the desire for food, educating them that any impulses will be transitory and can be tolerated	
- Encouraging patients to consider their efforts to control eating as a necessary condition for achieving healthy weight loss and benefiting from its associated physical and psychological advantages	
- Involving patients actively in identifying processes hindering weight loss using the “Weight-Loss Obstacles Questionnaire”	
- Developing collaboratively with the patients their personal formulation of the processes that are hindering weight loss	
- Designing personalized procedures aimed at addressing the specific obstacles encountered by each patient	
- Involving, with the consent of patients, their significant others in treatment to create the optimal environment for facilitating patients attempts efforts to change their eating habits	
• *Strategies and procedures for improving weight-loss maintenance*	
- Addressing weight-loss satisfaction before starting weight-loss maintenance	
- Dedicating one or two sessions to preparing patients for weight maintenance, and collaboratively developing a weight maintenance plan	
- Encouraging patients to suspend any attempts to lose weight while learning weight-maintenance skills (i.e., at least 12 months)	
- Creating a list of personal reasons to maintain weight	
- Adopting a mindset with a constant focus on weight control, and keeping a constant but flexible focus on weight control and self-awareness regarding diet and physical activity	
- Identifying and addressing high-risk weight- regain situations, preventing lapses from becoming relapses, and addressing any weight regain	
- Implementing weekly self-weighing and ensuring patients maintain weight within a specific range of 4 kg	
- Encouraging patients to follow a high-protein, low-glycaemic-index diet with moderate fat content, and to practice at least 30 min of moderate-intensity activity daily	

As such, CBT-OB places great emphasis on engaging the patients in the treatment, partly by encouraging them to prioritize treatment and play an active role in replacing their unhealthy habits with those conducive to weight control. To this end, the generic CBT strategies of real-time self-monitoring and carrying out strategic homework tasks are adopted, although other elements of generic CBT, such as formal thought records, schemas, assumptions, and automatic thoughts, are not introduced. Although CBT-OB does address some aspects of cognitive bias, such as generalization and dichotomous thinking, as CBT-E [18], it does not often rely on complex cognitive restructuring, but instead seeks to promote cognitive change (i.e., a change of their frame of mind) with simpler means by encouraging patients to analyse the effects and implications of strategic and achievable modifications to their behaviour. Both in and outside sessions, patients are asked to observe their own behaviour and seek to identify anything that is standing in the way of their adopting a lifestyle that will help them lose, and subsequently maintain, weight. The therapist aims to stimulate a patient’s interest in the effects and implications of trying different ways of behaving. Then, when they consistently implement the new eating and physical activity habits and demonstrate a persistent “weight-*control* mindset”, they are helped to identify triggers that are likely to reactivate their “weight-*gain* mindset”, to recognize the first signs that this is occurring, and to take preventative action straight away (to “do the right thing”, generally the opposite of the behaviour driven by the weight-gain mindset). In this way, patients learn to manipulate their own frame of mind [19], and thereby deal more effectively with weight gain by immediately averting setbacks that might otherwise develop into full-scale relapses.

Other adaptations include a higher frequency of sessions in the first weeks of treatment, when the focus is on helping patients to “start well” placing great emphasis on establishing and maintaining therapeutic momentum (i.e., identifying the best time for patients to start CBT-OB, stressing the importance of avoiding any interruptions during the treatment, and planning one session a week for the first eight weeks of the treatment) as early weight loss has been shown to be a good predictor of long-term weight loss [20]. In addition, a subgroup of patients with severe and disabling obesity may begin treatment in intensive settings, such as day-hospital or residential CBT-OB units [21], to boost their chances of success.

Further details of CBT-OB, together with a comprehensive description of the treatment and its implementation, can be found in the main treatment guide [15]. In addition to reading the manual, the therapist delivering the treatment should attend a workshop given by an expert in CBT-OB providing an overview of the intervention and its strategies and procedures. However, it is also advisable that therapists receive supervision in implementing the treatment from someone proficient in it.

### The versions of CBT-OB

**Fig. 2:**
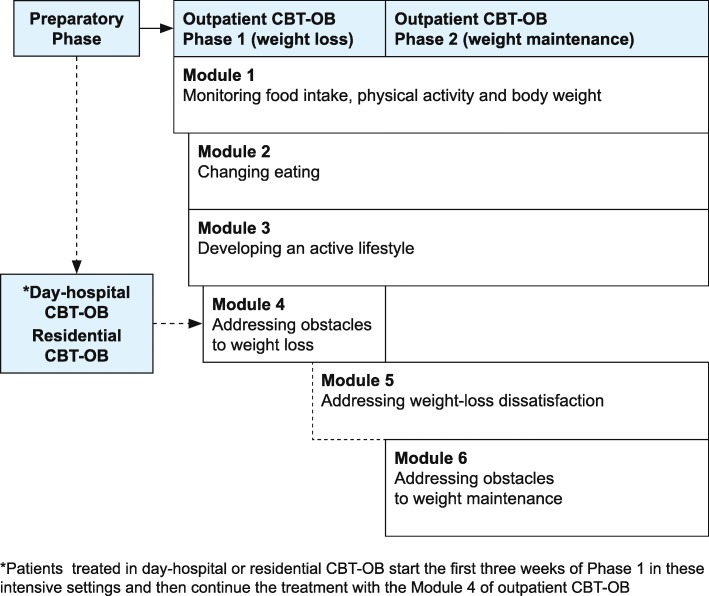
The map of cognitive behavioural therapy for obesity (CBT-OB) From Dalle Grave et al. [15], 20. Reprinted with the permission of Springer Nature

CBT-OB has been designed to treat all classes of obesity within a stepped-care approach involving three levels of care (outpatient, day-hospital and residential). Outpatient CBT-OB can be delivered individually by a single therapist or in group by two therapists. It includes the following phases (see Fig. 2):

- *Preparatory Phase.* This is delivered in one or two sessions, and has the aims of assessing the nature and severity of a patient’s obesity, as well as any associated medical and psychosocial comorbidities, as well as engaging the patient(s) in the treatment.
- *Phase 1.* This has been designed to help patients achieve a healthy rate of weight loss and be satisfied with the resulting weight. It lasts about 24 weeks and is delivered across 16 sessions, the first eight of which are held once a week, and the remaining eight on a two-weekly basis.
- *Phase 2*. This has the aim of helping patients to develop a lifestyle and mindset conducive to long-term weight maintenance. It usually lasts 48 weeks and is delivered across 12 sessions that are held at four-weekly intervals.

CBT-OB is delivered in six modules (see Table 3), each including specific strategies and procedures that may be adapted to the patient’s individual progress and barriers; the six modules are introduced in a flexible and individualized way, according to the patient’s needs, across Phase 1 and Phase 2. In general, however, Module 1 is introduced in the first session, Module 2 and 3 in the second session, Module 4 in the third session, Module 5 when the patient reports dissatisfaction with weight loss unrelated to poor adherence to the diet and exercise programme, and Module 6 at the beginning of Phase 2.

**Table 3:** The main procedures of the six CBT-OB modules

*Module 1 - Monitoring food Intake, physical activity and body weight*	
• Initiating weekly weighing	
• Explaining what the treatment will involve	
• Educating on energy balance	
• Establishing real-time monitoring of food intake and physical activity	
• Initiating weekly weighing	
*Module 2 - Changing eating*	
• Creating an energy deficit of 500–1000 kcal per day produce a variable weight loss of about 0.5–1 kg a week.	
• Planning ahead when, what and where to eat	
• Eating consciously	
*Module 3 - Developing an active lifestyle*	
• Assessing the patient’s eligibility for exercise	
• Assessing the patient’s functional exercise capacity	
• Motivating the patient to exercise	
• Developing an active lifestyle, reducing sedentary activities and increasing the daily step count	
• Improving physical fitness	
• Continuing or commencing formal exercise (in selected cases)	
*Module 4 - Addressing obstacles to weight loss*	
• Educating the patients on cognitive-behavioural weight-loss obstacles (antecedent stimuli, positive consequences, problematic thoughts)	
• Introducing the Weight-Loss Obstacles Questionnaire	
• Creating the Personal Formulation	
• Addressing weight-loss obstacles	
- Reducing environmental stimuli	
- Addressing events influencing eating and exercise habits	
- Addressing impulses and emotions influencing eating and exercise habits	
- Addressing problematic thoughts	
- Addressing the use of food as a reward, and the patient’s rational excuses for not adopting an active lifestyle	
*Module 5 - Addressing weight-loss dissatisfaction*	
• Detecting weight-loss dissatisfaction and its reasons	
• Addressing unrealistic weight goals	
• Addressing dysfunctional primary goals for losing weight	
• Addressing negative body image	
*Module 6: Addressing the obstacles to weight maintenance*	
• Reviewing the changes achieved through weight loss	
• Educating the patient on weight maintenance	
• Involving the patient actively in the decision to start weight maintenance	
• Introducing the procedures for weight maintenance	
- Establishing weekly self-weighing and a weight-maintenance range	
- Adopting eating habits and physical activity habits conducive to weight maintenance	
- Constructing a weight-maintenance mindset	
- Identifying and addressing high-risk situations and	
- Addressing weight regain	
• Discontinuing real-time monitoring of food intake	
• Evaluating possible future weight-loss attempts	
• Preparing a weight-maintenance plan	
• Bringing the treatment to a close	

Each session lasts 45 min (90 min when the treatment is delivered in group), and is divided into five parts, each with a distinct objective, specifically:

1. In-session collaborative weighing (up to 5 min)
2. Reviewing self-monitoring and other homework (up to 10 min)
3. Collaboratively setting the session agenda (about 2 min)
4. Working through the agenda and agreeing on homework tasks (up to 30 min).
5. Concluding the session (about 3 min). This includes summarizing what has been addressed in session, confirming the homework assignment(s), and scheduling the next appointment.

Day-hospital and residential CBT-OB, on the other hand, which are indicated for patients with severe and disabling obesity with no upper limit of body mass index (BMI) [22], last 21 days. A distinctive characteristic of these intensive versions is they are delivered by a multidisciplinary CBT-OB-trained team of physicians, dieticians, psychologists, physiotherapists and nurses, all acting in concert. Relying on the same principles as outpatient CBT-OB, intensive versions of the treatment include the following main procedures [23]: (i) a low-energy diet; (ii) a motor/functional rehabilitation programme; and (iii) a daily group CBT-OB session in which patients are actively trained to use the procedures outlined in Modules 1–3 (as in outpatient CBT-OB). Day-hospital CBT-OB is similar to residential CBT-E, but the patients sleep in their home. After discharge, patients are advised to continue CBT-OB treatment in the outpatient setting. Since such patients have already implemented the procedures of Modules 1–3 (generally delivered during the first two sessions of outpatient CBT-OB) during the intensive CBT-OB phase, these can be omitted from the “post-intensive” outpatient CBT-OB (see Fig. 1).

CBT-OB may be also associated with weight loss drugs and/or bariatric surgery in selected cases [24], or in some cases followed by these interventions if there is a need of further weight loss, and can also be adapted for patients with obesity associated with binge-eating disorder (BED) [25].

Finally, CBT-OB is contraindicated for patients who are pregnant or lactating, take medication affecting body weight, have medical comorbidities associated with weight loss or have severe psychiatric disorders (e.g., major depression, acute psychotic disorders, substance use disorders, and bulimia nervosa).

### CBT-OB effectiveness

A randomized control trial has assessed the effectiveness of CBT-OB in 88 patients with severe obesity, allocated either a high-protein diet (HPD) or a high-carbohydrate diet (HCD) [26]. The treatment studied in this trial included 3 weeks of residential CBT-OB followed by outpatient CBT- OB. Encouragingly, the attrition rate observed in the both HPD (25.6%) and HCD (17.8%) groups was far lower than the 50% attrition rate commonly observed in the community after standard biomedical prescriptive weight-loss treatments [27] and similar to that reported by research trials of BT-OB [12]. Furthermore, weight loss at 43 weeks in completers (*n* = 69) was 15% for HPD and 13.3% for HCD, a percentage weight loss which was much higher than the mean 8% in 6 months reported by conventional lifestyle-modification programmes based on BT-OB [26]. No significant difference between the two arms was observed throughout the trial, and both diets produced similar improvements in cardiovascular risk factors and psychological profiles; what is more, no tendency to regain weight was observed in either group between 6 and 12 months [26].

Another study, conducted in an Italian National Health Service obesity clinic, assessed the effectiveness of group CBT-OB in 67 patients with severe obesity. In this case the treatment was less intensive than recommended by the CBT-OB protocol, including only 22 group sessions (14 in the 6-month weight-loss phase and 8 in the subsequent 12-month weight-maintenance phase) [21]. Nevertheless, 76.2% patients completed the treatment, displaying an average weight loss of 11.5% after 6 months and 9.9% after 18 months. This weight loss was associated with a significant reduction in cardiovascular risk factors, anxiety, depression and eating-disorder psychopathology, and an improvement in obesity-related quality of life [21].

Another study compared the long-term effects of residential CBT-OB in 54 patients with severe obesity with or without binge-eating disorder (BED) [28]. Even though patients did not receive outpatient CBT-OB after discharge, at 5-year follow-up, 51.5% of the former group no longer met the diagnostic criteria for BED. Moreover, no difference was observed between the two groups in terms of mean weight loss (6.3 kg in BED vs. 7.4 kg in non-BED).

Finally, one observational outpatient study on CBT-OB delivered individually in a real-world clinical setting is ongoing.

## Conclusions

CBT-OB is an innovative treatment designed to help patients maintain long-term weight loss by addressing some limitations of traditional BT-OB, namely the poor personalization of the intervention and the prevalent focus on helping the patients to reach behavioural change (i.e., eating and exercise habits) rather than a cognitive change oriented to long-term weight control. As such, CBT-OB includes the main procedures of traditional BT-OB, but includes new strategies and procedures, introduced according to the individual patient’s needs, to address specific cognitive processes that previous research has found to be associated with treatment discontinuation, weight loss and weight maintenance. Moreover, it can be delivered in a stepped-care approach, including three levels of care (i.e., outpatient, day-hospital, and residential) to treat patients with severe and disabling obesity. If the promising results displayed by CBT-OB so far are confirmed by future randomized controlled trials comparing CBT-OB with BT-OB, the treatment has the potential to improve on the outcomes achievable by traditional lifestyle-modification weight-loss treatments.

## Data Availability

Not applicable.

## Abbreviations

BT-OB: Behavioural-therapy for obesity
CBT: Cognitive-behavioural therapy
CBT-E: Enhanced-cognitive behavioural therapy for eating disorders
CBT-OB: Cognitive-behavioural therapy for obesity
OB: Obesity
